# Comparative Effectiveness and Safety of High-Intensity Focused Ultrasound for Uterine Fibroids: A Systematic Review and Meta-Analysis

**DOI:** 10.3389/fonc.2021.600800

**Published:** 2021-03-09

**Authors:** Yi Wang, Jinsong Geng, Haini Bao, Jiancheng Dong, Jianwei Shi, Qinghua Xi

**Affiliations:** ^1^Department of Radiology, Zhongshan Hospital, Fudan University, Shanghai, China; ^2^Ministry of Education Virtual Research Center of Evidence-Based Medicine at Nantong University, Medical School of Nantong University, Nantong, China; ^3^Shanghai Jiaotong University School of Medicine, Shanghai, China; ^4^Affiliated Hospital of Nantong University, Nantong, China

**Keywords:** high intensity focused ultrasound (HIFU), uterine fibroids, meta-analysis, myomectomy, uterine arterial embolisation

## Abstract

**Background:** Uterine fibroids are common benign tumors among premenopausal women. High- intensity focused ultrasound (HIFU) is an emerging non-invasive intervention which uses the high-intensity ultrasound waves from ultrasound probes to focus on the targeted fibroids. However, the efficacy of HIFU in comparison with that of other common treatment types in clinical procedure remains unclear.

**Objective:** To investigate the comparative effectiveness and safety of HIFU with other techniques which have been widely used in clinical settings.

**Methods:** We searched the Cochrane Central Register of Controlled Trials, PubMed, EMBASE, Cumulative Index to Nursing & Allied Health Literature, Web of Science, ProQuest Nursing & Allied Health Database, and three Chinese academic databases, including randomized controlled trials (RCTs), non-RCTs, and cohort studies. The primary outcome was the rate of re-intervention, and the GRADE approach was used to interpret the findings.

**Results:** About 18 studies met the inclusion criteria. HIFU was associated with an increased risk of re-intervention rate in comparison with myomectomy (MYO) [pooled odds ratio (OR): 4.05, 95% confidence interval (CI): 1.82–8.9]. The results favored HIFU in comparison with hysterectomy (HYS) on the change of follicle-stimulating hormone [pooled mean difference (MD): −7.95, 95% CI: −8.92–6.98), luteinizing hormone (MD: −4.38, 95% CI: −5.17−3.59), and estradiol (pooled MD: 43.82, 95% CI: 36.92–50.72)]. HIFU had a shorter duration of hospital stay in comparison with MYO (pooled MD: −4.70, 95% CI: −7.46−1.94, *p* < 0.01). It had a lower incidence of fever (pooled OR: 0.15, 95% CI: 0.06–0.39, *p* < 0.01) and a lower incidence of major adverse events (pooled OR: 0.04, 95% CI: 0.00–0.30, *p* < 0.01) in comparison with HYS.

**Conclusions:** High-intensity focused ultrasound may help maintain feminity and shorten the duration of hospital stay. High-quality clinical studies with a large sample size, a long-term follow-up, and the newest HIFU treatment protocol for evaluating the re-intervention rate are suggested to be carried out. Clinical decision should be based on the specific situation of the patients and individual values.

## Introduction

Uterine fibroids are common benign tumors which are rich in extracellular matrix among premenopausal women (1, 2). Fibroids can cause severe menstrual bleeding and menorrhagia, which may lead to iron deficiency anemia. Large fibroids can lead to pelvic pain and pressure on the rectum with painful or difficult defecation. Fibroids are the potential causes of recurrent miscarriages (3, 4). The conventional surgical approaches to fibroid treatment comprise hysterectomy (HYS) or abdominal myomectomy (MYO) for those desiring uterine preservation. Physicians are seeking new ways to treat uterine fibroids, which may allow patients to avoid invasive surgery. Minimally invasive techniques for the treatment of uterine fibroids have been developed in recent years, such as high-intensity focused ultrasound (HIFU), laparoscopic MYO, uterine artery embolization (UAE), and radiofrequency ablation (RFA) (5–7).

High-intensity focused ultrasound is an emerging intervention which uses the high-intensity ultrasound waves from ultrasound probes to focus on the targeted fibroids. It is a non-invasive technique that causes instant coagulated necrosis in a well-circumscribed area of a few millimeters in diameter and can be performed under the guidance of either MRI or ultrasound. HIFU has been increasingly performed in China and has now become a preferred therapy of uterine fibroids in some hospitals, especially for women with fibroid-associated bulk symptoms who desire for uterine-sparing and fertility-sparing surgeries.

Recent studies have compared the effectiveness of HIFU with that of some treatment techniques. Nevertheless, the results obtained from individual studies are sometimes contradictory. At present, the comparative benefits and risks of HIFU for the treatment of uterine fibroids remain unclear. The objective of the present systematic review and meta-analysis is to evaluate the comparative effectiveness and safety of HIFU in the treatment of uterine fibroids. We specifically aimed to compare HIFU with different techniques which have been widely used in clinical practice.

## Materials and Methods

### Search Strategy and Study Selection

Trials were identified by searching the Cochrane Central Register of Controlled Trials (CENTRAL), PubMed, EMBASE, Cumulative Index to Nursing & Allied Health Literature (CINAHL), Web of Science, and ProQuest Nursing & Allied Health Database. A search of Chinese academic databases, including Wanfang Data, VIP Chinese Science and Technique Journals Database (VIP-CSTJ), and China National Knowledge Infrastructure (CNKI), was also carried out. Among the studies published in Chinese journals, only the journals indexed by the ExLibris Chinese Core Journal Searching System were considered to reduce publication bias. The protocol of this systematic review has been registered at PROSPERO (No. CRD42018115773). Databases were searched on July 8, 2020. A detailed search strategy was given (Appendix 1). There were no limitations to languages of the included studies. Reference lists were examined for any additional relevant studies which were not identified through the search. Randomized controlled trials (RCTs), non-RCTs, and cohort studies were included in the review.

Two review authors (Wang Y and Geng JS) independently screened all titles and abstracts of publications identified by the search to assess their eligibility. We excluded at this stage the publications that did not meet the criteria. Following the screening, we assessed the full texts of eligible citations for inclusion. We reached a consensus on the selection of trials and the final list of studies. We consulted a third member of our review team (Dong JC) when a consensus could not be reached. The inclusion criteria for the full text were observational studies, RCTs, or non-RCTs published in English before July 8, 2020, which provide data on the clinical assessment of outcomes of patients with uterine fibroids after being treated with HIFU and the other clinically used techniques as comparison groups. In order to build a more comprehensive database concerning this subject, there is a need for the inclusion of publications in Chinese or other languages in our study. The following criteria were used for exclusion: (1) reviews, conference abstracts, case reports, opinions, and comments; (2) patients having undergone earlier treatment for uterine fibroids; (3) no outcome of interest was found; and (4) no suitable data (no standard deviation or interquartile range) can be used for statistical analysis.

### Eligibility Criteria

- Types of participants: Women with a definite diagnosis of uterine fibroids, regardless of age, were included. Patients who had previous intervention for fibroids were excluded;
- Types of intervention and comparison: Both MRI-guided HIFU (MRIgHIFU) and ultrasound-guided HIFU (USgHIFU) were included. Comparison groups comprised of techniques other than HIFU, which were usually used in clinical practice, such as UAE, RFA, MYO (including laparoscopy MYO), and HYS.

### Data Extraction

The following metrics were extracted from the eligible articles: (a) study characteristics: first author name, publication date, participant factors (patient's age, number, fibroid size), trial design, details of intervention and control, and follow-up information; (b) primary outcome: the rate of re-intervention after using HIFU or comparative techniques; and (c) secondary outcomes: defining the incidence of abnormal pregnancy in the abnormal pregnancy percentage for those patients with uterine fibroids who got pregnant after the treatment with HIFU or comparative techniques. The change of serum sex hormones, including follicle-stimulating hormone (FSH), luteinizing hormone (LH), and estradiol (E2), was assessed. The days of hospital stay for patients with uterine fibroids during the treatment period were calculated. The incidence of complications and adverse events were noted. Significant clinical complications were defined as fever within 2–3 days after the treatment, and the incidence of patients experiencing at least one major adverse event within 6 weeks after the treatment. Studies without having any of the abovementioned outcomes were excluded from the meta-analysis.

### Assessment of Risk of Bias

The risk of bias of RCTs was assessed according to “The Cochrane Collaboration's tool” (8). The characteristics of RCTs were evaluated as follows: randomization, allocation concealment, blinding of outcome assessment, incomplete outcome data, and selective reporting. The risk of bias of non-RCTs was assessed according to “Methodological Index for Non-randomized Studies” (MINOS) (9). We deleted the item “‘endpoints appropriate to the aim of the study” from MINOS since not every end point of the published papers was included in our systematic review. Therefore, the remaining 11 characteristics of non-RCTs were evaluated. Cohort studies were assessed in accordance with a “Newcastle-Ottawa Quality Assessment Scale” (NOS) (10). Characteristics of cohort studies including the selection of cohorts, comparability of cohorts, and the outcomes were evaluated.

The GRADE approach (11) was used to interpret findings for the primary outcomes, and the GRADE profile allowed us to import data from Review Manager 5.3 to create “Summary of findings” tables. We downgraded the evidence quality from “high quality” by one level for serious (or by two for very serious) study limitations (risk of bias), inconsistency, indirectness of evidence, imprecision of effect estimates, or potential publication bias.

### Data Synthesis and Statistical Analysis

Data synthesis and statistical analysis were presented using Review Manager 5.3 (RevMan, Review Manager 5.3). We used odds ratio (OR) with 95% confidence interval (CI) for dichotomous data. We used mean difference (MD) with 95% CI for continuous data when data were provided as mean and SD. The heterogeneity of intervention has effects among the studies using the standard Chi-square test (*p*-value). We considered a value of *p* < 0.10 as evidence of heterogeneity (8). In case of no substantial or considerable heterogeneity, we utilized a fixed-effects model in the data synthesis. Otherwise, we used a random-effects model. Moreover, a subgroup analysis was designed for each outcome according to the different types of comparisons. In addition, a sensitivity analysis was performed to evaluate the stability of this meta-analysis, and an intention-to-treat analysis was conducted when available to test the robustness of the results. Finally, the Begg–Mazumdar's rank test and the Egger's regression test were used to assess publication bias, in which a value of *p* < 0.05 was considered statistically significant. We only evaluated the publication bias of fever rate, since the number of included studies of other outcomes was less than seven, which was considered not sufficient for an analysis.

## Results

### Study Characteristics

**Figure 1:**
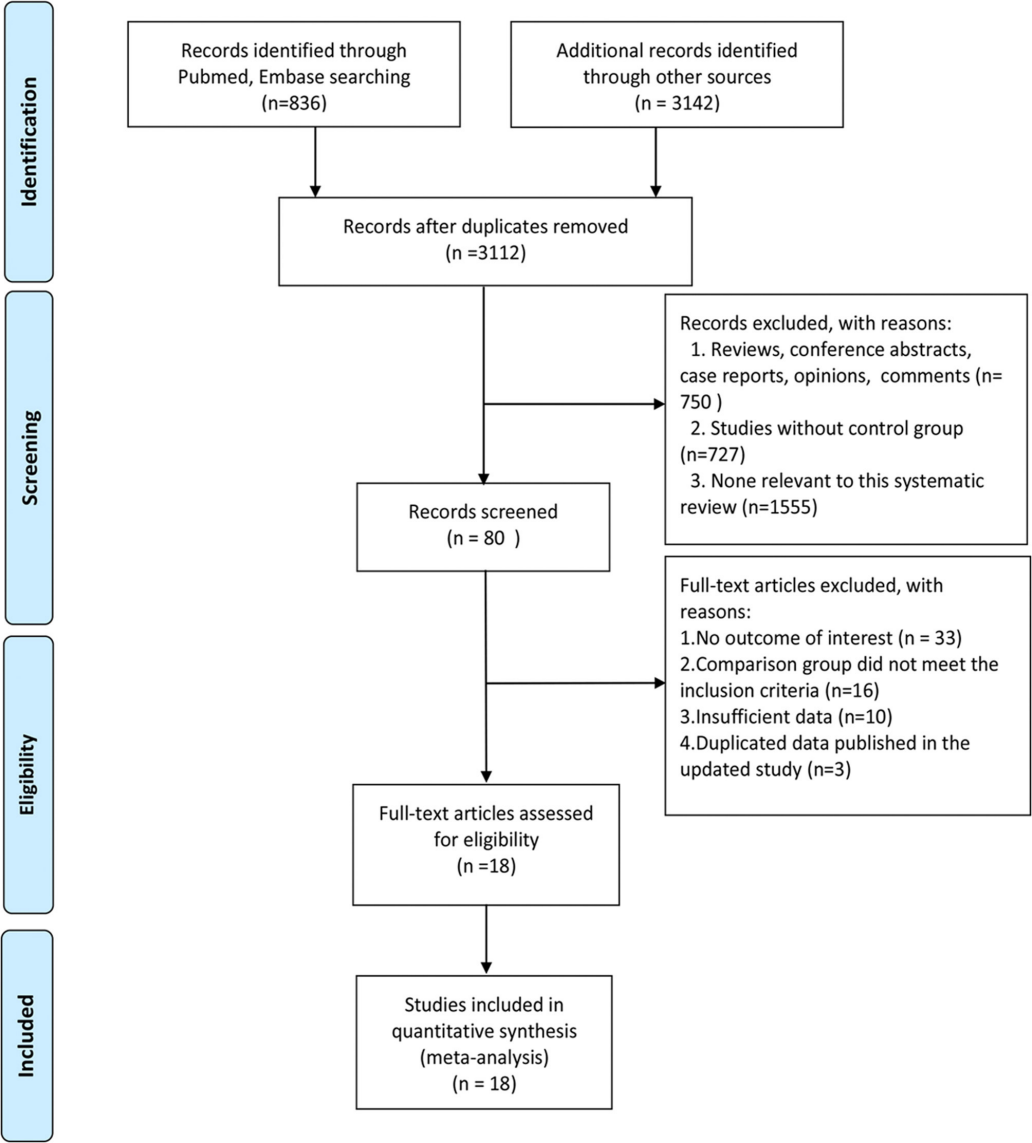
A PRISMA FlowChart of study selection.

About 18 studies were included in the review (12–27). About three were RCTs (16, 18, 21), seven were non-RCTs (12, 15, 17, 22–24, 26), and eight were cohort studies (13, 14, 19, 20, 25, 27–29). In Barnard et al. (19), only the cohort data were included in the meta-analysis. One study (17) was carried out in 14 medical centers in USA, Israel, UK, and Germany, and the other studies were conducted in China (*n* = 13), USA (*n* = 1), Germany (*n* = 1), The Netherlands (*n* = 1), and Israel (*n* = 1). The average age of women who participated in the included studies ranged from 33.60 to 46.54 years. PRISMA Flow diagram of study selection is listed in Figure 1. Characteristics of included studies are listed in Table 1.

**Table 1:** Characteristics of included studies.

**References**	**Study design**	**Setting**	**No. of patients**	Age of participants (years)^*^	**Manufactures of HIFU**	**Outcomes of interests (duration of follow-up after treatment)**
Taran et al. (17)	Non-RCT	14 medical centers in USA, Israel, UK and Germany	HIFU:109 HYS: 83	HIFU:44.8 ± 4.9; HYS:44.4 ± 5.6	InSightec	Fever (2 days)
Meng et al. (16)	RCT	China	HIFU: 50 RFA: 50	HIFU:35.6 ± 6.0; RF:39.2 ± 5.7	Shanghai Aishen Technology	Fever (2 days)
Chen et al. (12)	Non-RCT	China	HIFU: 30 MYO(abdominal/laparoscopic MYO):30 HYS: 30	HIFU:38.8; MYO:38.4; HYS:39.1	Chongqing Haifu Technology	Change of serum sex hormones (6 months)
Froeling et al. (13)	Retrospectivecohort study	Germany	HIFU: 36 UAE: 41	HIFU:36.2(29.2–41.0); UAE:42.7(33.6–52.2)	InSightec	Rate of re-intervention (60.7–61.9 months)
Liu et al. (15)	Non-RCT	China	HIFU: 30 HYS: 30	HIFU:39.25 ± 3.08; HYS:41.13 ± 3.22	Chongqing Haifu Technology	Change of serum sex hormones (1 year)
Wang et al. (18)	RCT	China	HIFU: 60 MYO (abdominal MYO): 60	HIFU:39.92 ± 5.07; MYO:38.60 ± 4.36	Chongqing Haifu Technology	Fever (3 days)
Ikink et al. (14)	Retrospective cohort study	Netherlands	HIFU: 51 UAE: 68	HIFU:46(43–49); UAE:43(41–46)	Philips Healthcare	Rate of re-intervention (1 year)
Wang et al. (23)	Non-RCT	China	HIFU: 89 MYO (laparoscopic MYO): 41	HIFU:37.9 ± 5.5; MYO:38.4 ± 5.0	Chongqing Haifu Technology	Days of hospital stay; Fever (2 days); Major adverse events (42 days)
Wang et al. (24)	Non-RCT	China	HIFU: 86 HYS: 81	HIFU:33.6 ± 4.6; HYS:34.1 ± 4.7	Chongqing Haifu Technology	Fever (2 days); Major adverse events (42 days)
Xu et al. (26)	Non-RCT	China	HIFU: 30 MYO (laparoscopic MYO): 34	Both groups:37.7	Chongqing Haifu Technology	Change of serum sex hormones (6 months)
Barnard et al. (19) ^*^	Prospective cohort study	USA	HIFU: 43 UAE: 40	HIFU:44.0 ± 4.3; UAE:44.3 ± 5.2	InSightec	Rate of re-intervention (42 days); Major adverse events (42 days)
Lin et al. (22)	Non-RCT	China	HIFU: 60 UAE: 54	HIFU:39.5 ± 7.4; UAE: 38.7 ± 6.2	Chongqing Haifu Technology	Incidence of abnormal pregnancy (2 years); Change of serum sex hormones (2 years); Fever (2 days)
Xiong et al. (25)	Retrospective cohort study	China	HIFU: 206 MYO (laparoscopic MYO): 317	HIFU:41.37 ± 5.68; MYO:40.48 ± 5.58	Chongqing Haifu Technology	Rate of re-intervention (1.5–4 years); Incidence of abnormal pregnancy (1.5–4 years); Days of hospital stay
Chen et al. (20)	Prospective cohort study	China	HIFU: 1,353 MYO (abdominal/laparoscopic MYO): 586 HYS: 472	HIFU:41.31 ± 5.08; MYO:40.93 ± 5.02; HYS:46.54 ± 3.48	Chongqing Haifu Technology	Rate of re-intervention (1 year); Days of hospital stay; Fever (2 days); Major adverse events (30 days)
Li et al. (21)	RCT	China	HIFU: 60 MYO (laparoscopic MYO): 60	HIFU:38.4 ± 5.4; MYO:39.3 ± 6.82	Chongqing Haifu Technology	Incidence of abnormal pregnancy (3 years); Days of hospital stay
Mohr-Sasson et al. (27)	Retrospective cohort study	Israel	HIFU: 68 MYO^#^(laparoscopic MYO): 64	HIFU:44(38–47); MYO:38(34–43)	InSightec	Rate of re-intervention (31–36 months)
Wu et al. (28)	Retrospective cohort study	China	HIFU: 219 MYO (laparoscopic MYO): 224	HIFU:31.6 (22–42); MYO:32.4 (25–41)	Chongqing Haifu Technology	Incidence of abnormal pregnancy (1–8 years)
Hu et al. (29)	Prospective cohort study	China	HIFU: 39 MYO (hysteroscopic MYO): 42	HIFU:43.0 ± 5.6 MYO:41.3 ± 4.4	Chongqing Haifu Technology	Days of hospital stay

*HIFU, high intensity focused ultrasound; UAE, uterine artery embolization; MYO, myomectomy; HYS, hysterectomy; RFA, radio frequency ablation*.

* *Mean ± SD or Median(range)*.

# *In the myomectomy group, 29 women (45.3%) received robotically assisted laparoscopic myomectomy (Da Vinci Surgical system)*.

About three RCTs with unclear risk of bias were included in the systematic review. For seven non-RCTs, the average score was 15.4 according to MINOS, indicating the moderate quality. Blinding was not used in the non-RCTs, and some objective outcomes, such as abnormal pregnancy and serum sex hormones, were unlikely to be influenced by the lack of blinding. However, according to MINOS, only the reported and adequate items could be given two scores. Seven of the eight included cohort studies were equal to or more than six stars according to NOS, which suggested a moderate quality. Detailed information on the summary of the risk of bias is presented in Supplementary Tables 1–3.

### Meta-Analysis

#### Primary Outcomes

##### Rate of Re-intervention

**Figure 2:**
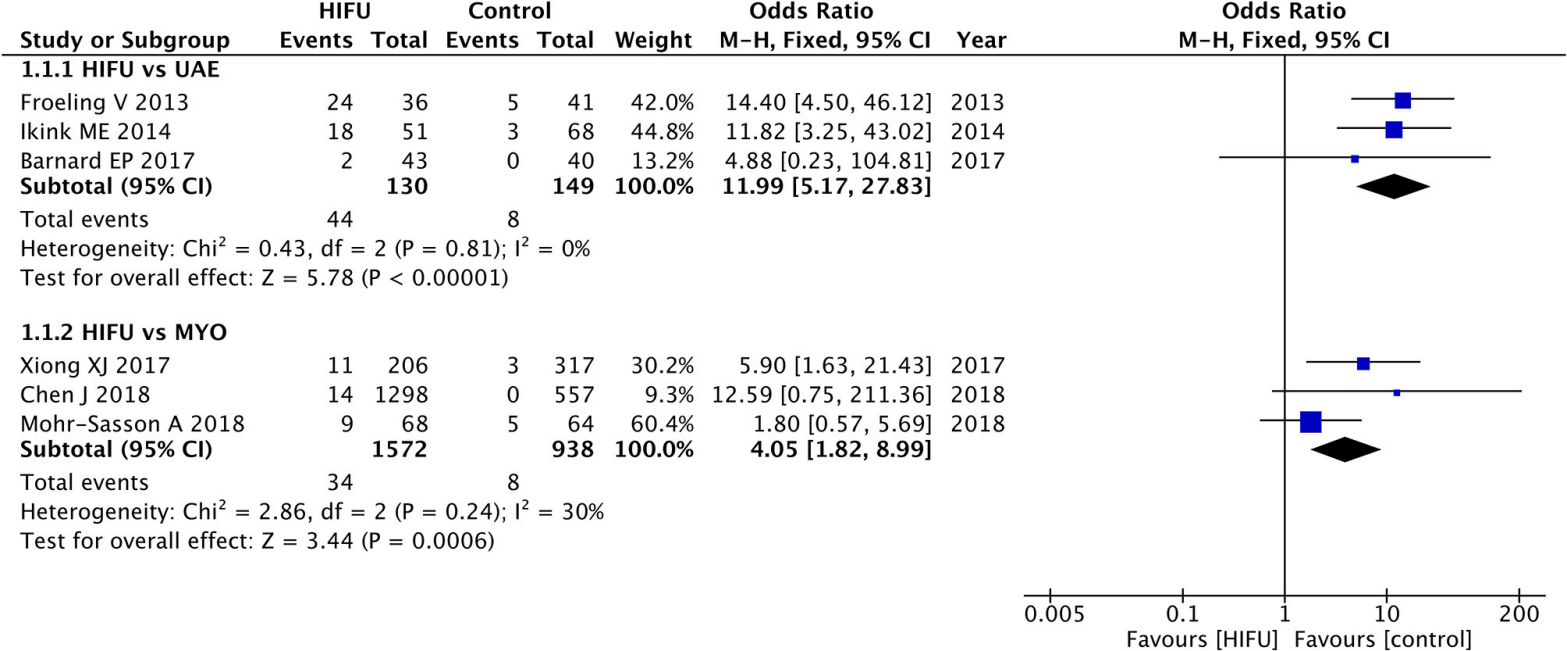
The re-intervention rate of HIFU in comparison with other techniques.

High-intensity focused ultrasound was associated with an increased risk of re-intervention rate in comparison with UAE (pooled OR: 11.99, 95% CI: 5.17–27.83, *p* < 0.01) and MYO (pooled OR: 4.05, 95% CI: 1.82–8.99, *p* < 0.01) (Figure 2).

The results from the intention-to-treat analysis also found an increased risk of re-intervention for HIFU in comparison with UAE (pooled OR: 9.33, 95% CI: 4.26–20.47, *p* < 0.01) and MYO (pooled OR: 4.51, 95% CI: 2.02–10.07, *p* < 0.01) (Supplementary Figure 1). The sensitivity analysis did not change the increased re-intervention rate for HIFU in comparison with UAE and MYO (Supplementary Figure 2).

The GRADE evidence profile is given in Supplementary Table 4, which lists the results of relative effects and absolute effects. The overall quality of evidence regarding HIFU vs. UAE and HIFU vs. MYO was moderate and low, respectively.

#### Secondary Outcomes

##### Incidence of Abnormal Pregnancy

**Figure 3:**
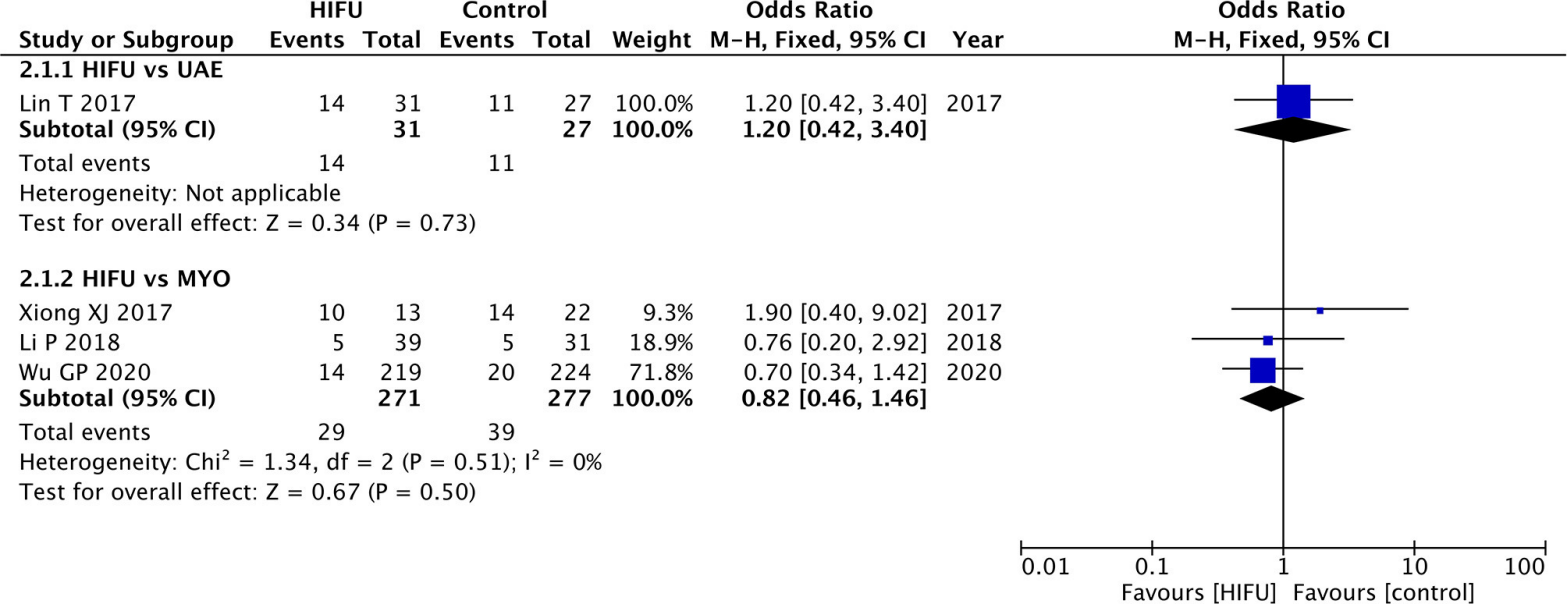
Abnormal pregnancy incidence of HIFU in comparison with other techniques.

Lin et al. (22) analyzed the incidence of abnormal pregnancy between HIFU and UAE. The results obtained from this study did not find any statistical differences between these two techniques (OR: 1.20, 95% CI: 0.42–3.40, *p* = 0.73) (Figure 3).

Statistically significant differences were not found when comparing HIFU with MYO (pooled OR: 0.82, 95% CI: 0.46–1.46, *p* = 0.50). The results obtained from the intention-to-treat analysis did not present any change to the conclusion (Supplementary Figure 3).

##### Change of Serum Sex Hormones From Baseline

There were no statistically significant differences between HIFU and MYO on the FSH, LH, and E2 levels (Table 2, Supplementary Figures 4–6).

**Table 2:** Meta-analysis of serum sex hormones change between HIFU and MYO.

**Outcome measure**	**Number of studies**	**Test for heterogeneity (*P*-value)**	**Pooled MD (95% CI)**	**Test for overall effect (*P*-value)**
FSH	2 (12, 26)	0.93	0.00 (−0.58 0.59)	0.99
LH	2 (12, 26)	0.65	−0.11 (−0.73 0.51)	0.73
E2	2 (12, 26)	0.61	1.14 (−3.29 5.57)	0.24

However, HIFU seemed to be better in terms of maintaining the serum FSH, LH, and E2 levels in comparison with HYS (Table 3, Supplementary Figures 4–6).

**Table 3:** Meta-analysis of serum sex hormones change between HIFU and HYS.

**Outcome measure**	**Number of studies**	**Test for heterogeneity (*P*-value)**	**Pooled MD (95% CI)**	**Test for overall effect (*P*-value)**
FSH	2 (12, 26)	0.60	−7.95 (−8.92 −6.98)	<0.01
LH	2 (12, 26)	0.02	−4.38 (−5.17 −3.59)	<0.01
E2	2 (12, 26)	0.73	43.82 (36.92 50.72)	<0.01

Furthermore, Lin et al. (22) compared the change of serum sex hormones between HIFU and UAE. Data from this study did not find any statistically significant differences between these two techniques for FSH (MD: −0.20, 95% CI: −0.91–0.51, *p* = 0.58), LH (MD: 0.10, 95% CI: −0.55–0.75, *p* = 0.76), and E2 (MD: −1.00, 95% CI: −7.42–5.42, *p* = 0.76) (Supplementary Figures 4–6).

##### Days of Hospital Stay

**Figure 4:**
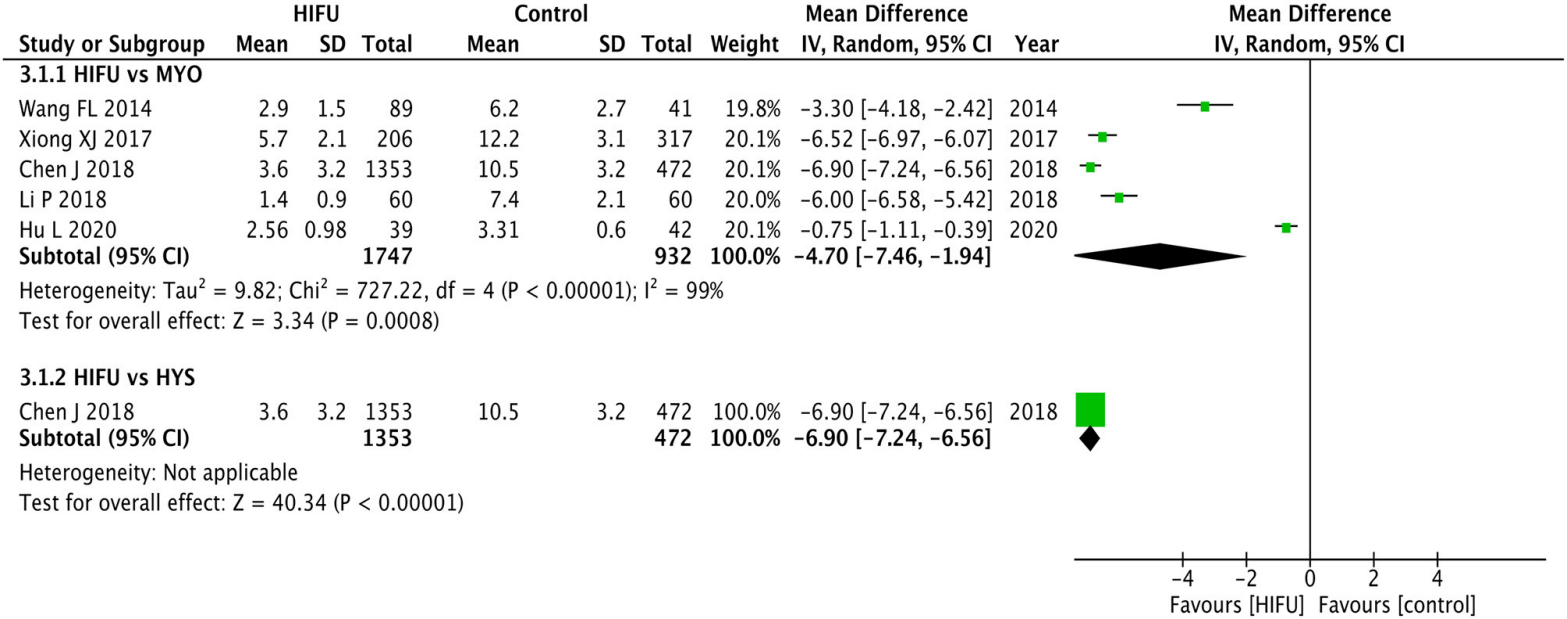
Hospital stay of HIFU in comparison with other techniques.

Although statistical heterogeneity was found for the included studies (*p* < 0.01, *I*^2^ = 93%) (Figure 4), the direction of the individual studies remains the same and the results of meta-analysis favored the shorter duration of hospital stay for HIFU in comparison with MYO (pooled MD: −4.70, 95% CI: −7.46−1.94, *p* < 0.01).

The results from the included study (20) also favored HIFU in comparison with HYS (MD: −6.90, 95% CI: −7.24−6.56, *p* < 0.01).

### Incidence of Complications and Adverse Events

#### Incidence of Fever

**Figure 5:**
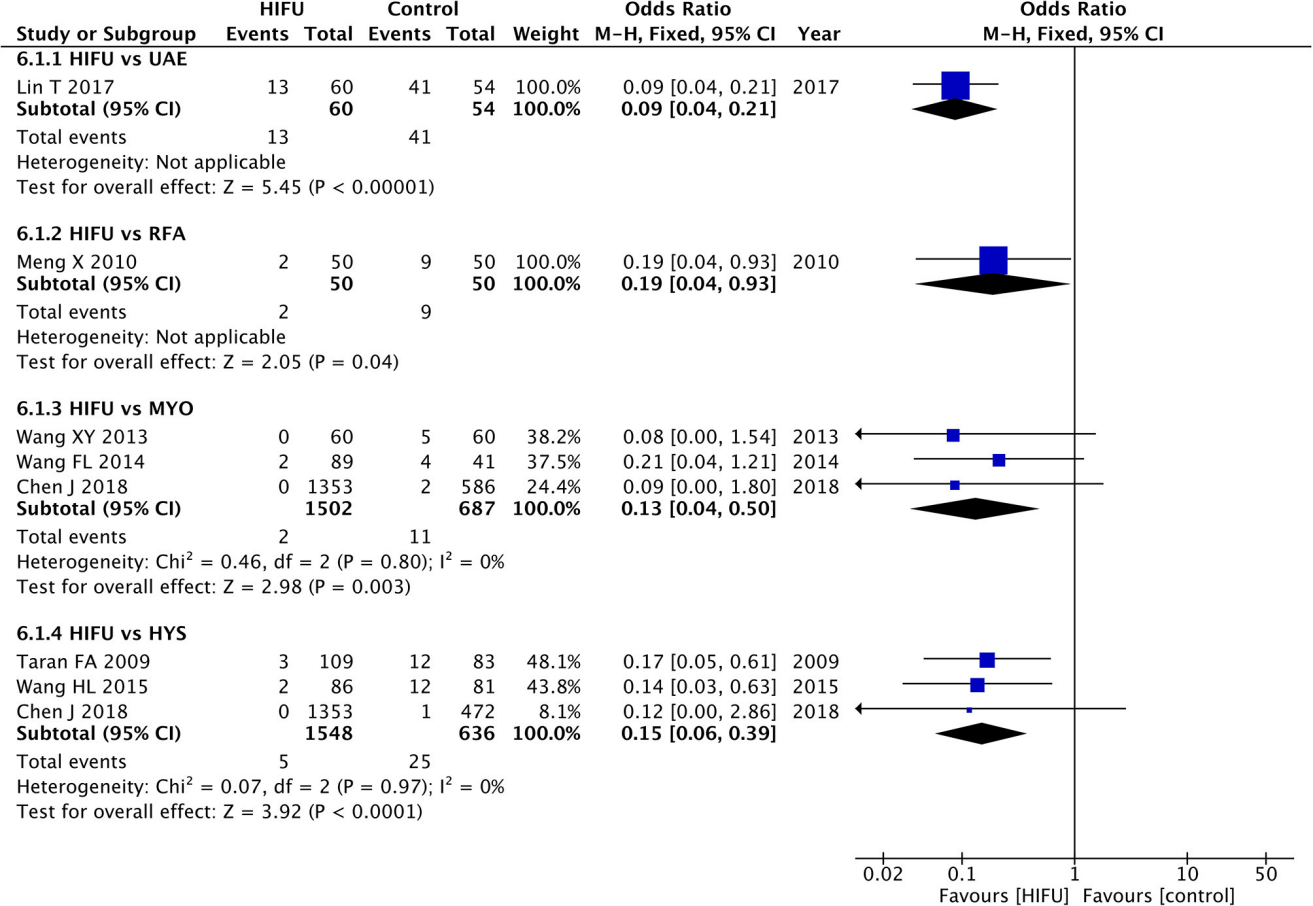
Fever incidence of HIFU in comparison with other techniques.

Meta-analysis showed a lower incidence of fever in HIFU in comparison with MYO (pooled OR: 0.13, 95% CI: 0.04–0.50, *p* < 0.01); HIFU could also decrease the incidence of fever in comparison with HYS (pooled OR: 0.15, 95% CI: 0.06–0.39, *p* < 0.01) (Figure 5).

The sensitivity analysis showed the beneficial effects of HIFU in comparison with HYS (Supplementary Figure 7). However, only one study (23) was included in the sensitivity analysis to compare the incidence of fever between HIFU and MYO, and no significant statistical differences were identified.

Lin et al. (22) investigated the incidence of fever in HIFU in comparison with UAE, and the results favored HIFU (OR: 0.09, 90% CI: 0.04–0.21, *p* < 0.01). The results obtained from the study by Meng et al. (16) favored HIFU in comparison with RFA (OR: 0.19, 90% CI: 0.04–0.93, *p* = 0.04).

#### Incidence of Major Adverse Events

**Figure 6:**
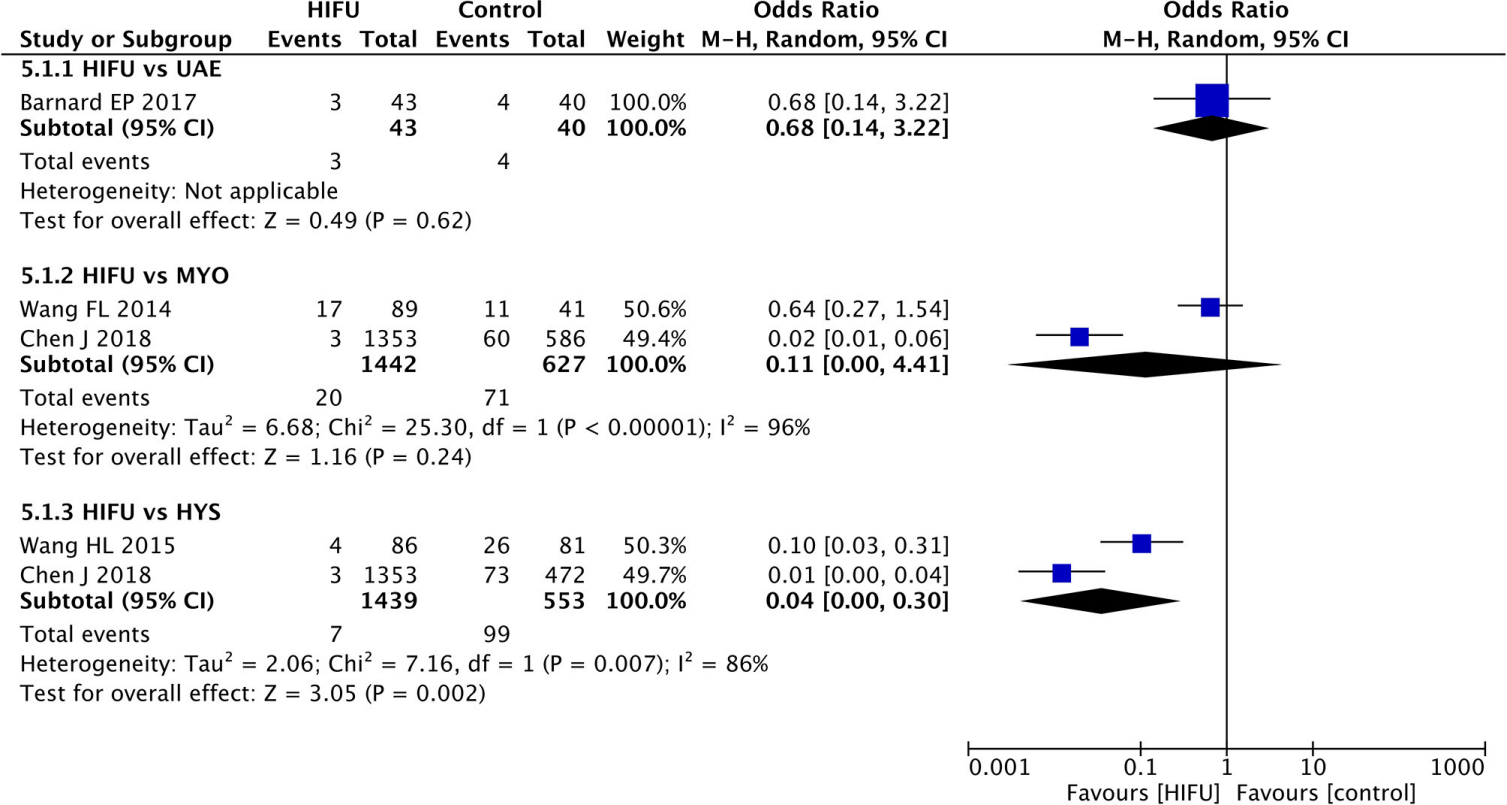
Major adverse events of HIFU in comparison with other techniques.

The results of the meta-analysis did not found statistical significant differences between HIFU and MYO (pooled OR: 0.11, 95% CI: 0.00–4.41, *p* < 0.01) (Figure 6). However, significant heterogeneity among the included studies was identified (*p* < 0.01).

The meta-analysis favored the lower incidence of major adverse events in HIFU in comparison with HYS (pooled OR: 0.04, 95% CI: 0.00–0.30, *p* < 0.01). Although heterogeneity of the included studies was identified (*p* < 0.01), the results of the individual studies were consistent with those of the meta-analysis.

No statistical significant difference was identified between HIFU and UAE from the included study (19) (OR: 0.68, 95% CI: 0.14–3.22, *p* = 0.62).

#### Assessment of Bias Across Included Studies

No significant publication bias was observed in the outcome of fever rate (Begg–Mazumdar's test: *p* = 0.81, Egger's test: *p* = 0.58).

## Discussion

Uterine fibroids are the most common tumors of the female reproductive tract. Due to lack of the latest published synthesized evidence on primary studies on the relative efficacy and safety of the different types of treatment techniques, choosing the best option for a patient might sometimes become difficult. When counseling a patient about the different treatment options, the re-intervention risk is a crucial aspect to consider. We demonstrated that HIFU had the least promising outcome regarding the re-intervention incidence in comparison with UAE and MYO. Our results are consistent with another meta-analysis (30), which showed that the cumulative risk of re-intervention for UAE is 14.4% and for HIFU is 54% at 60 months after initial therapy. Previous studies (31–33) have demonstrated that the non-perfused volume (NPV) ratio is an optimal predictor of re-intervention rates, and the fibroids with NPVs more than 50–60% were less likely to need additional treatments. Verpalen et al. (33) conducted subgroup analyses of HIFU stratified by treatment protocols and found that the re-intervention rates in an unrestrictive protocol of HIFU are significantly lower than those of a restrictive protocol after a 7-years follow-up. However, the studies included in the review did not classify patients from the HIFU group according to characteristics of fibroids and a specific HIFU treatment option. Hence, the outcomes of our study should be interpreted cautiously, which possibly cause an underlying confoundingresult. In the future, high-quality clinical studies should be implemented to achieve more specific results to guarantee further modification of treatment protocol after evaluating the individual condition. Wang et al. (34) compared the efficacy of HIFU and other uterine-sparing surgeries for the treatment of submucosal fibroids with an deep intramural extension, concluding that HIFU had lower re-intervention rates. Simon et al. (35) implemented a novel HIFU treatment using a modified energy transmission and oxytocin augmentation, which resulted in lower re-intervention rates in comparison with UAE. Moreover, a few studies (36, 37) pointed out that the ablation effect of HIFU could be enhanced by using a microbubble contrast agent, which could significantly increase the post-operative NPV ratio and reduce the incidence of recurrence of uterine fibroids.

Hysterectomy is the most common treatment for symptomatic fibroids and is considered to be the definitive therapy. HYS was recommended for premenopausal women who had no wish to preserve their fertility (38). However, childbearing-age women with multiple submucosal and intramural fibroids presented with menorrhagia and pelvic pain also desire future pregnancies and was concerned about the loss of femininity. To date, MYO remains the gold standard for treating fibroid-related symptoms in women who desire fertility preservation. It was recommended by the guideline that MYO might be considered to optimize pregnancy outcomes in women with asymptomatic cavity-distorting myomas (39). In a systematic review (40), MYO has higher successful pregnancy rates (75.6%) in comparison with UAE (60.6%), which might be explained by a high risk of disruption of blood supply to the ovary and intima after the UAE treatment. Liu et al. (41) reported that a successful vaginal delivery rate after the HIFU ablation had reached 80.8%. Other studies (28, 42) reported that pregnancy outcomes of HIFU were not compromised, and in comparison with laparoscopic MYO, HIFU is conducive to decreasing cesarean delivery rate since HIFU focuses on the lesion without damaging the surrounding normal tissue of the uterine. On the contrary, conventional surgery would have a high risk of pelvic cavity adhesion. However, HIFU has a higher incidence of preterm birth, through fetal distress.

Lee et al. (43) and Cheung et al. (44) assessed the changes in anti-mullerian hormone levels after the ablation of uterine fibroids, which also showed that HIFU did not impair ovarian function. We found that HIFU presented an obviously advantage over HYS with significantly less fluctuations in hormonal mediators, and it is non-inferior to UAE and MYO. However, the results should be interpreted with caution, since two of the included studies (12, 26) analyzed the hormone levels only at 6 months after the treatment, and only one study (22) was included to compare the change of serum sex hormones between HIFU and UAE.

High-intensity focused ultrasound is associated with fewer adverse events; therefore, the duration of hospital stay is shorter. The reduction of the days of hospital stay has widely attracted attention from policymakers as an important way to improve efficiency and quality of medical care (45, 46). High cost effectiveness of the protocol can largely alleviate the economic and social burden. Nevertheless, there are no current studies reporting the days of hospital stay by comparing HIFU and other minimally invasive therapies like UAE or RFA.

Patients have different preferences in regard to surgical procedures and the potential risk of adverse outcomes. Therefore, we analyzed the incidence of serious adverse events. HIFU was also more favorable in comparison with MYO and HYS on the incidence of major adverse events after the treatment. In a multicenter large cohort study (47), a total of 0.408% major complications of HIFU have been observed, while in another study (48), laparoscopic MYO had an incidence of 3.5% significant complications. Meanwhile, it is worth noting that Verpalen et al. (49) demonstrated that there is a significant difference between Sonalleve (17.6%) and Exblate (5.7%) when evaluating the rate of adverse events.

Evidence for the use of HIFU in our systematic review was mainly Available online at non-randomized studies, and robust evidence of evaluating comparative efficacy and safety of HIFU for the treatment of uterine fibroids in these studies remains lacking. However, data from cohort studies and non-RCTs could not be underestimated, since these studies can better mimic the real clinical setting in comparison with RCTs (50–52). Furthermore, RCTs are not always feasible to be conducted in certain clinical circumstances. RCTs, non-RCTs, and cohort studies are valuable to determine more accurate outcomes in clinical practice.

## Conclusion

This study provides clinicians with latest published comparative evidence between HIFU and other widely used clinical treatment methods. Currently, patients usually prefer less invasive options for the treatment regardless of pregnancy. Our results found that HIFU seemed to be safer and more effective than HYS. HIFU was non-inferior to MYO in maintaining the serum sex hormone levels, as well as the prevention of abnormal pregnancy, and was more effective than MYO in reducing the incidence of fever. HIFU could become a potential efficient technique to shorten the duration of hospital stay. The comparative efficacy and safety of HIFU with other types of minimally invasive techniques, such as UAE, still deserve to be further assessed. High-quality clinical studies with a large sample size and a long-term follow-up are suggested to be performed in future to further evaluate the re-intervention rate of HIFU, utilizing the advanced treatment protocol and equipment in comparison with the other treatment techniques.

It is crucial to realize the function of these treatment options in various clinical scenarios so that appropriate consultation could be performed. Patients should be informed of potential benefits and harms and should be actively involved in the choice of surgery. The treatment decision depends on the clinical symptoms the desire of patients for subsequent fertility and pregnancy, as well as efficacy and need for repeated interventions.

## Data Availability Statement

The original contributions presented in the study are included in the article/Supplementary Material, further inquiries can be directed to the corresponding author/s.

## Disclosure

National Natural Science Foundation of China (Grant No. 71603138) was used for this research project. The authors declare that the research was conducted in the absence of any commercial or financial relationships that could be construed as a potential conflict of interest.

## Author Contributions

YW, JG, and HB contributed to the conception and design of the study. HB and QX were responsible for data acquisition and interpretation. YW and QX performed the statistical analysis. YW and JG wrote sections of the manuscript. All authors contributed to manuscript revision, read, and approved the submitted version.

## Conflict of Interest

The authors declare that the research was conducted in the absence of any commercial or financial relationships that could be construed as a potential conflict of interest.

## Supplementary Material

Supplementary Figure 1 Re-intervention rate of high-intensity focused ultrasound (HIFU) in comparison with other techniques: intention to treat (ITT) analysis.

Supplementary Figure 2 Re-intervention rate of HIFU in comparison with other techniques: sensitivity analysis.

Supplementary Figure 3 Abnormal pregnancy incidence of HIFU in comparison with other techniques: ITT analysis.

Supplementary Figure 4 Follicle-stimulating hormone (FSH) change of HIFU in comparison with other techniques.

Supplementary Figure 5 Luteinizing hormone (LH) change of HIFU in comparison with other techniques.

Supplementary Figure 6 Estradiol (E2) change of HIFU in comparison with other techniques.

Supplementary Figure 7 Fever incidence of HIFU in comparison with other techniques: sensitivity analysis.

Supplementary Table 1 Risk of bias of included randomized controlled trials.

Supplementary Table 2 Risk of bias of included non-randomized controlled trials. ^*^For outcomes which were included in the systematic review. The items were scored as 0 (not reported), 1 (reported but inadequate), or 2 (reported and adequate).

Supplementary Table 3 Risk of bias of included cohort studies. ^*^For outcomes which were included in the systematic review. ^#^ Study controlled for: number of fibroid tumors/volume of all fibroid tissues or the maximal fibroid diameter, and age of patients. A study could be awarded a maximum of one star for each numbered item within the “Selection” and “Outcome” categories, and a maximum of two stars could be given for “Comparability.”

Supplementary Table 4 GRADE evidence profile of the outcome of rate of re-intervention. 1. Cohort studies were assessed in accordance with the Newcastle-Ottawa Quality Assessment Scale (NOS), and the best quality of cohort studies should be those with nine stars. However, six stars were given to the three included studies. 2. The results from Barnard et al. (19), which only observed the short-term outcomes, did not find any statistical significant differences between HIFU and UAE. 3. Pooled odds ratio (OR) was 11.99 (95% confidence interval: 5.17–27.83, *p* < 0.001). 4. For Froeling et al. (13), volume of all fibroid tissues was significantly larger (*p* = 0.005) in the uterine artery embolization (UAE) group at baseline. However, in Ikink et al. (14), the maximum fibroid diameter and age at baseline were significantly higher (*p* < 0.005) in the MR-HIFU group. 5. Cohort studies were assessed in accordance with the NOS, and the best quality of cohort studies should be those with nine stars. Eight, seven, and five stars were given to the three included studies. 6. The results from Chen et al. (20) and Mohr-Sasson et al. (27) did not find any statistical significant differences between high-intensity focused ultrasound (HIFU) and myomectomy (MYO). 7. Pooled OR was 4.05 (95% CI 1.82–8.99, *p* < 0.001). 8. In Chen et al. (20), the mean age of the HIFU group was lower than that of the surgery group, and the uterine volume was smaller. In Mohr-Sasson et al. (27), women in the laparoscopic MYO group were younger (*p* < 0.001), and multiple uterine fibroid tumors were more common in the laparoscopic MYO group (*p* < 0.001).

## Abbreviations

HIFU: high-intensity focused ultrasound
UAE: uterine artery embolization
MYO: myomectomy
HYS: hysterectomy
RFA: radiofrequency ablation
FSH: follicle-stimulating hormone
LH: luteinizing hormone
E2: estradiol
MRIgHIFU: magnetic resonance image-guided HIFU
USgHIFU: ultrasound-guided HIFU.
