# Supplementary material for: Comparative Effectiveness and Safety of High-Intensity Focused Ultrasound for Uterine Fibroids: A Systematic Review and Meta-Analysis
Source: Front Oncol. 2021 Mar 9;11:600800. doi: 10.3389/fonc.2021.600800 (PMC7985460; doi:10.3389/fonc.2021.600800)
Supplement: Supplementary file 1 [file Data_Sheet_5.doc]

**Supplementary materials**

**Search strategy**

**Cochrane Central Register of Controlled Trials (CENTRAL)**

#1 MeSH descriptor: [Leiomyoma] explode all trees

#2 hysteromyoma or "uterine fibroid" or myoma or "uterine leiomyomata" or "uterine leiomyoma" or "uterine myomas" or "uterine myoma" or fibroid:ti,ab,kw

#3 "Magnetic resonance-guided focused ultrasound surgery" or MRgFUS or HIFU or "High-Intensity Focused Ultrasound Ablation" or "High Intensity Focused Ultrasound" or "Magnetic Resonance Guided Focused Ultrasound Surgery" or "Interventional Magnetic Resonance Imaging" or or focus?ed ultrasonograph* or "ultrasound-guided HIFU" or "USgHIFU" or focus?ed ultrasound* or insightec* or exablate*:ti,ab,kw

#4 #1 OR #2

#5 #3 AND #4

**PUBMED**

#1 "High-Intensity Focused Ultrasound Ablation"[Mesh]

#2 "High Intensity Focused Ultrasound"[Title/Abstract]

#3 "Magnetic Resonance Guided Focused Ultrasound Surgery"[Title/Abstract]

#4 Magnetic Resonance Imaging, Interventional[Title/Abstract]

#5 Interventional Magnetic Resonance Imaging[Title/Abstract]

#6 ((((MR?gFU*[Title/Abstract]) OR MR?g-FU*[Title/Abstract]) OR MR?gHIFU*[Title/Abstract]) OR MR?-HIFU*[Title/Abstract]) OR TcMR?gFU*[Title/Abstract] OR USgHIFU[Title/Abstract]

#7  (insightec*[Title/Abstract]) OR exablate*[Title/Abstract]

#8  (((((MRI[Title/Abstract]) OR MR[Title/Abstract]) OR MRI-guided[Title/Abstract]) OR magnetic resonance*[Title/Abstract]) OR unilateral[Title/Abstract]) OR high-intensity[Title/Abstract]

#9  (focus?ed ultrasound[Title/Abstract]) OR focus?ed ultrasonograph*[Title/Abstract]

#10 #8 AND#9

#11 #1 OR #2 OR #3 OR #4 OR #5 OR #6 OR #7 OR #10

#12 uterine fibroid[MeSH Terms]

#13 hysteromyoma[Title/Abstract] OR "uterine fibroid"[Title/Abstract] OR myoma[Title/Abstract] OR "uterine leiomyomata"[Title/Abstract] OR"uterine leiomyoma"[Title/Abstract] OR "uterine myomas"[Title/Abstract] OR "uterine myoma"[Title/Abstract] OR fibroid[Title/Abstract]

#14 #12 OR #13

#15 #11 AND #14

**EMBASE**

#1 'high intensity focused ultrasound'/exp

#2 ('interventional magnetic resonance imaging' or 'interventional ultrasonography' or 'high-intensity focused ultrasound ablation' or 'ultrasound therapy' or 'high intensity focused ultrasound'):ti,ab,kw

#3 focus?ed ultrasound*1 or focus?ed ultrasonograph*:ti,ab,kw

#4 MRI or MR or MRI-guided or MR-guided or magnetic resonance* or unilateral or high-intensity:ti,ab,kw

#5 #3 AND #4

#6 (MR?gFU* or MR?g-FU* or MR?gHIFU* or MR?-HIFU* or TcMR?gFU* or USgHIFU):ti,ab,kw

#7 insightec* or exablate*:ti,ab,kw

#8 #1 OR #2 OR #5 OR #6 OR #7

#9 'uterus myoma'/exp

#10 (uterine fibroid or hysteromyoma or uterine fibroid or myoma or uterine leiomyomata or uterine myomas or fibroid):ti,ab,kw

#11 #9 OR #10

#12 #8 AND #11

**Cumulative Index to Nursing & Allied Health Literature (CINAHL) (Search through EBSCO)**

#1 SU "High-Intensity Focused Ultrasound Ablation"

#2 AB "High Intensity Focused Ultrasound" OR AB "Magnetic Resonance Guided Focused Ultrasound Surgery" OR AB "Magnetic Resonance Imaging, Interventional" OR AB "Interventional Magnetic Resonance Imaging" OR AB "Magnetic Resonance Imaging, Interventional" OR AB "ultrasound-guided HIFU"

#3 AB insightec* OR AB exablate*

#4 AB "MRIgHIFU" OR AB "MRI HIFU" OR AB "MRI-HIFU" OR AB "USgHIFU" OR AB "HIFU"

#5  AB "MRI" OR AB "MR" OR AB "MRI-guided" OR AB magnetic resonance* OR AB unilateral OR AB "high-intensity"

#6 AB "focused ultrasound" OR AB focused ultrasonograph*

#7 #5 AND #6

#8 #1 OR #2 OR #3 OR #4 OR #7

**Web of Science**

#1 **TOPIC:** hysteromyoma OR "uterine fibroid" OR myoma OR "uterine leiomyomata" OR "uterine leiomyoma" OR "uterine myomas" OR "uterine myoma" OR fibroid

#2 **TOPIC:** "Magnetic resonance-guided focused ultrasound surgery" OR MRgFUS OR HIFU OR "High-Intensity Focused Ultrasound Ablation" OR "High Intensity Focused Ultrasound" OR "Magnetic Resonance Guided Focused Ultrasound Surgery" OR "Interventional Magnetic Resonance Imagin" OR "ultrasound-guided HIFU" OR "USgHIFU" OR focus?ed ultrasonograph* OR focus?ed ultrasound* OR insightec* OR exablate*

#3 #1 AND #2

Refined by: **[excluding]:Databases:** (MEDLINE)

**ProQuest Nursing & Allied Health Database**

#1 ab(hysteromyoma OR "uterine fibroid" OR myoma OR "uterine leiomyomata" OR "uterine leiomyoma" OR "uterine myomas" OR "uterine myoma" OR fibroid)

#2 ab("Magnetic resonance-guided focused ultrasound surgery" OR MRgFUS OR HIFU OR "High-Intensity Focused Ultrasound Ablation" OR "High Intensity Focused Ultrasound" OR "Magnetic Resonance Guided Focused Ultrasound Surgery" OR "Interventional Magnetic Resonance Imaging" OR"ultrasound-guided HIFU" OR "USgHIFU" OR focus?ed ultrasonograph* OR focus?ed ultrasound* OR insightec* OR exablate* )

#3 #1 AND #2
