# Supplementary figures and images for: Comparative Effectiveness and Safety of High-Intensity Focused Ultrasound for Uterine Fibroids: A Systematic Review and Meta-Analysis
Source: Front Oncol. 2021 Mar 9;11:600800. doi: 10.3389/fonc.2021.600800 (PMC7985460; doi:10.3389/fonc.2021.600800)

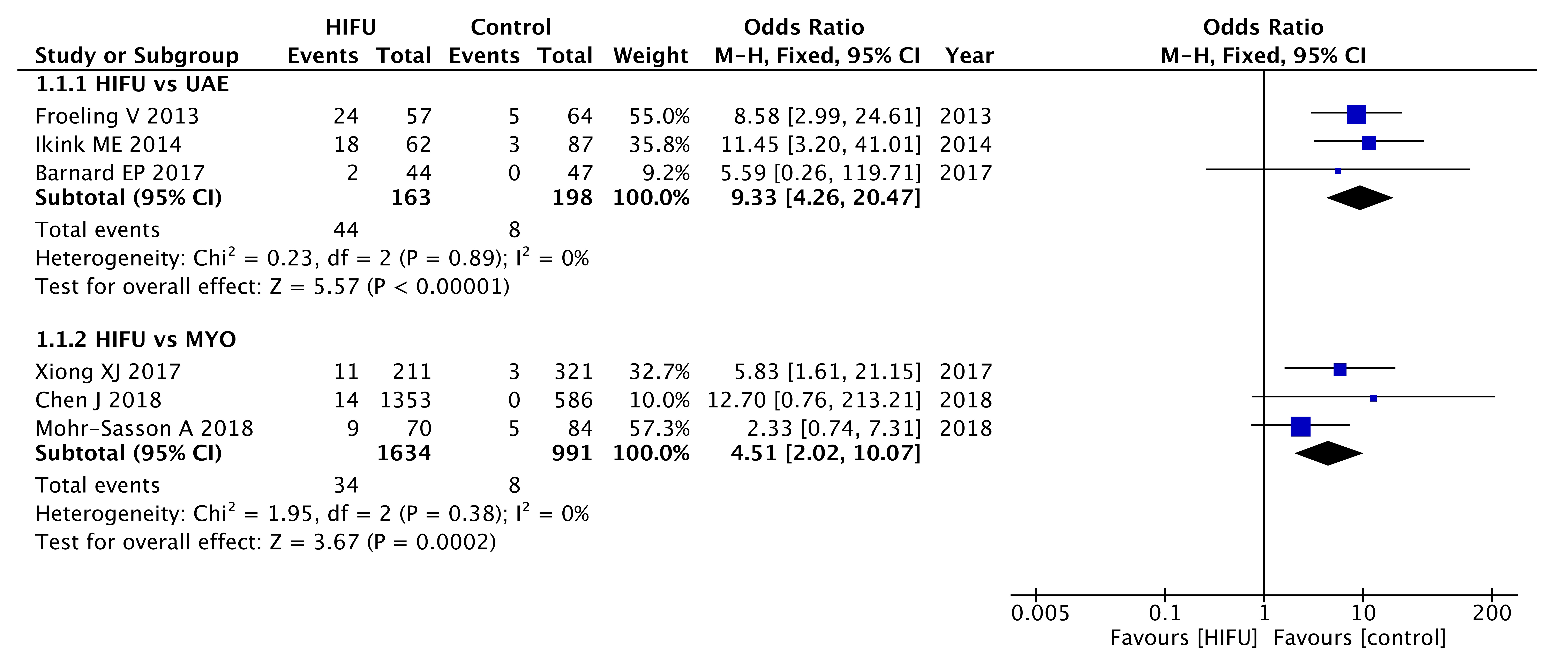

Supplement: Supplementary Figure 1 — Re-intervention rate of high-intensity focused ultrasound (HIFU) in comparison with other techniques: intention to treat (ITT) analysis. [file Image_1.TIF]

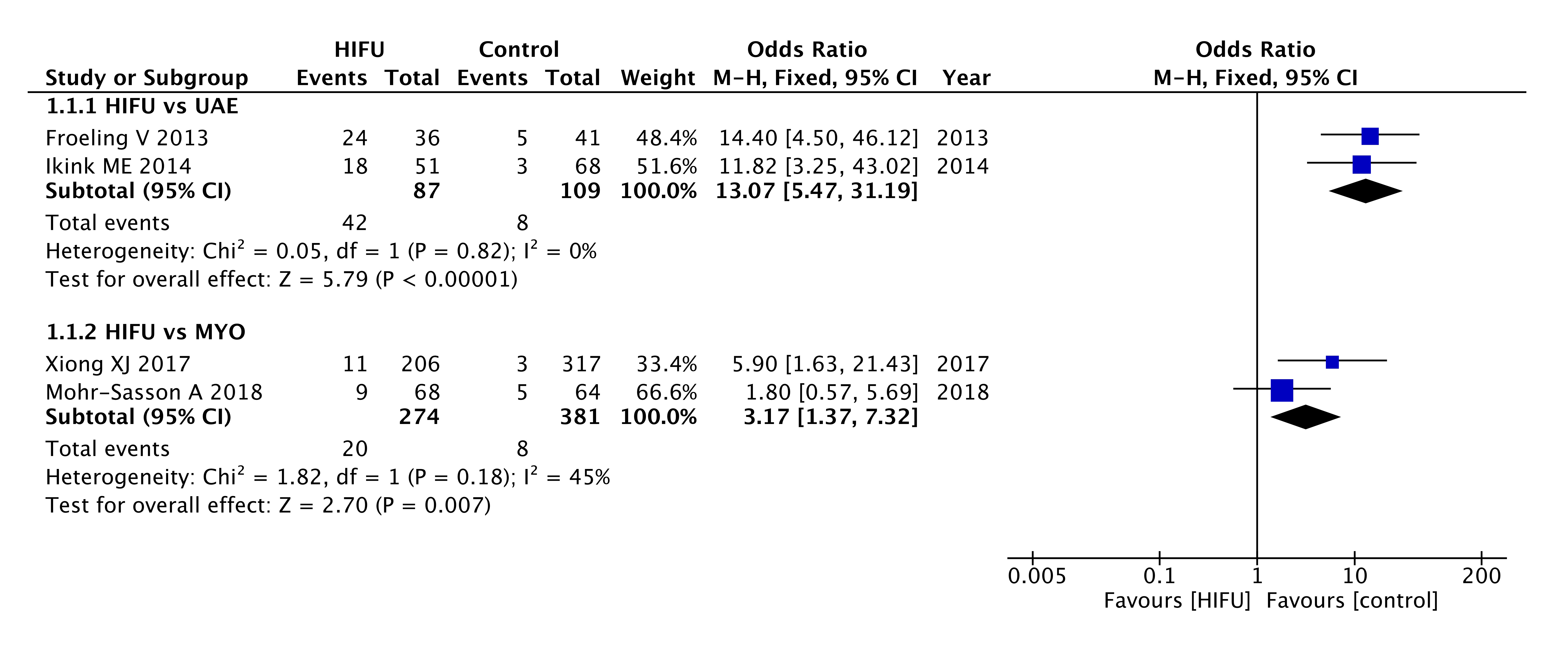

Supplement: Supplementary Figure 2 — Re-intervention rate of HIFU in comparison with other techniques: sensitivity analysis. [file Image_2.TIF]

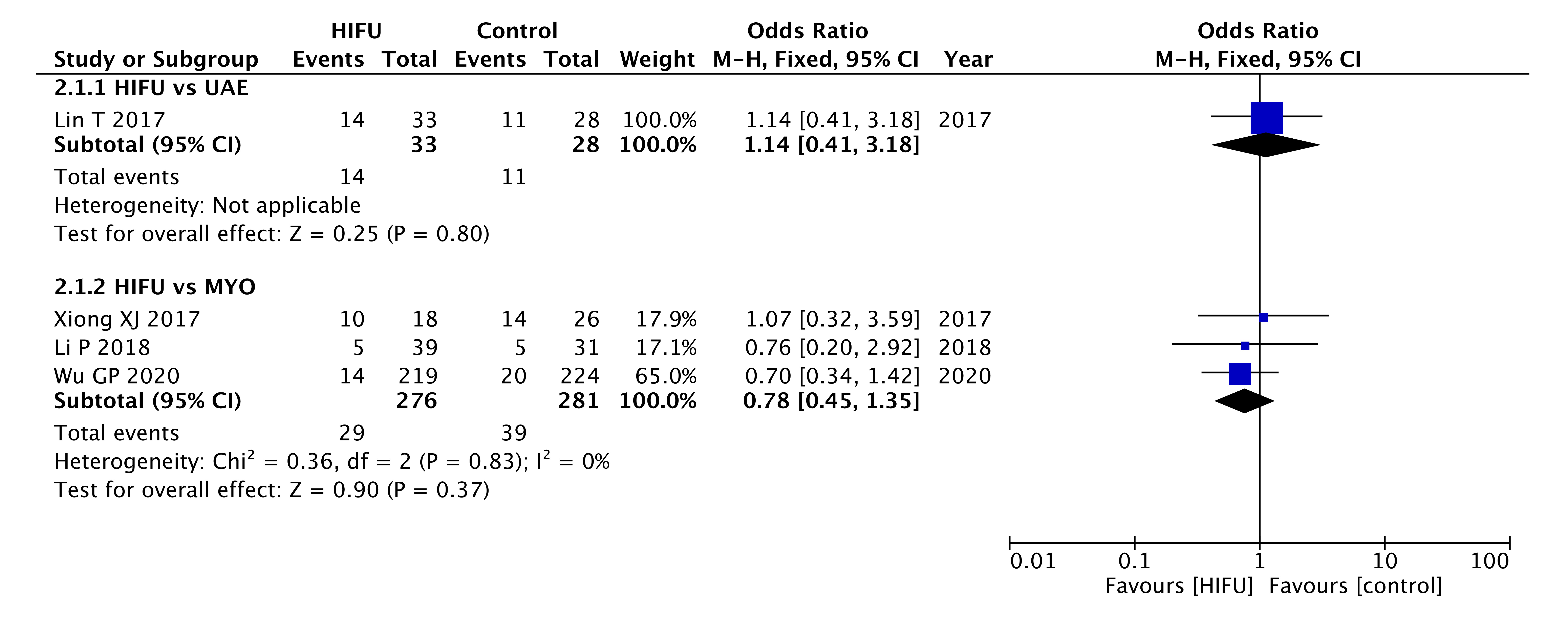

Supplement: Supplementary Figure 3 — Abnormal pregnancy incidence of HIFU in comparison with other techniques: ITT analysis. [file Image_3.TIF]

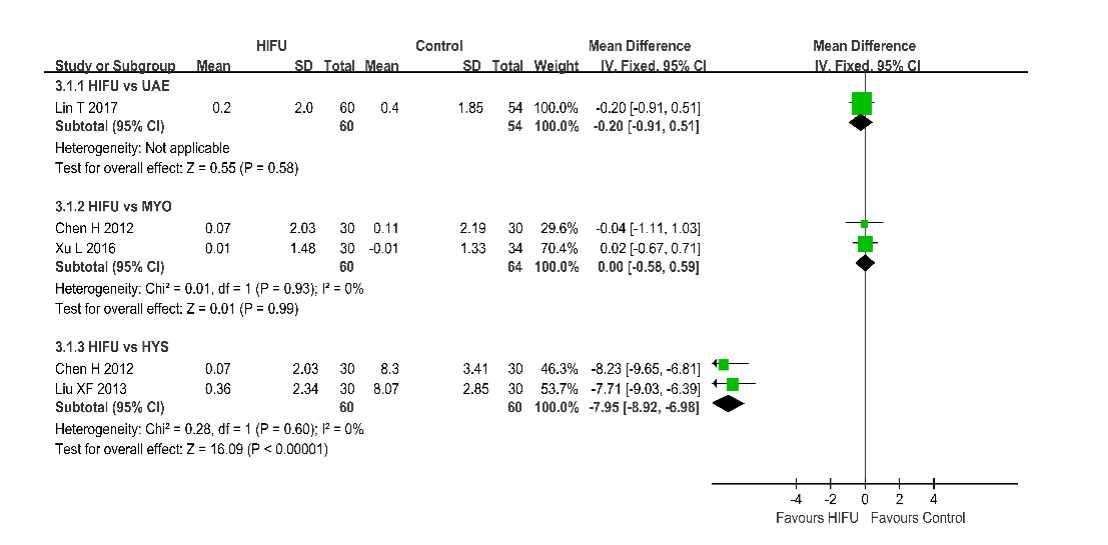

Supplement: Supplementary Figure 4 — Follicle-stimulating hormone (FSH) change of HIFU in comparison with other techniques. [file Image_4.TIF]

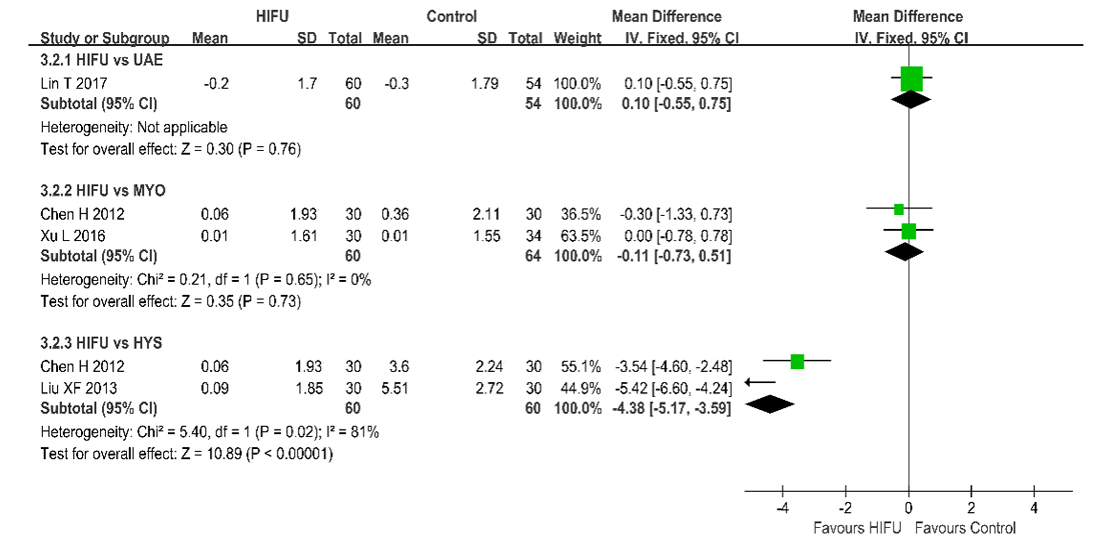

Supplement: Supplementary Figure 5 — Luteinizing hormone (LH) change of HIFU in comparison with other techniques. [file Image_5.TIF]

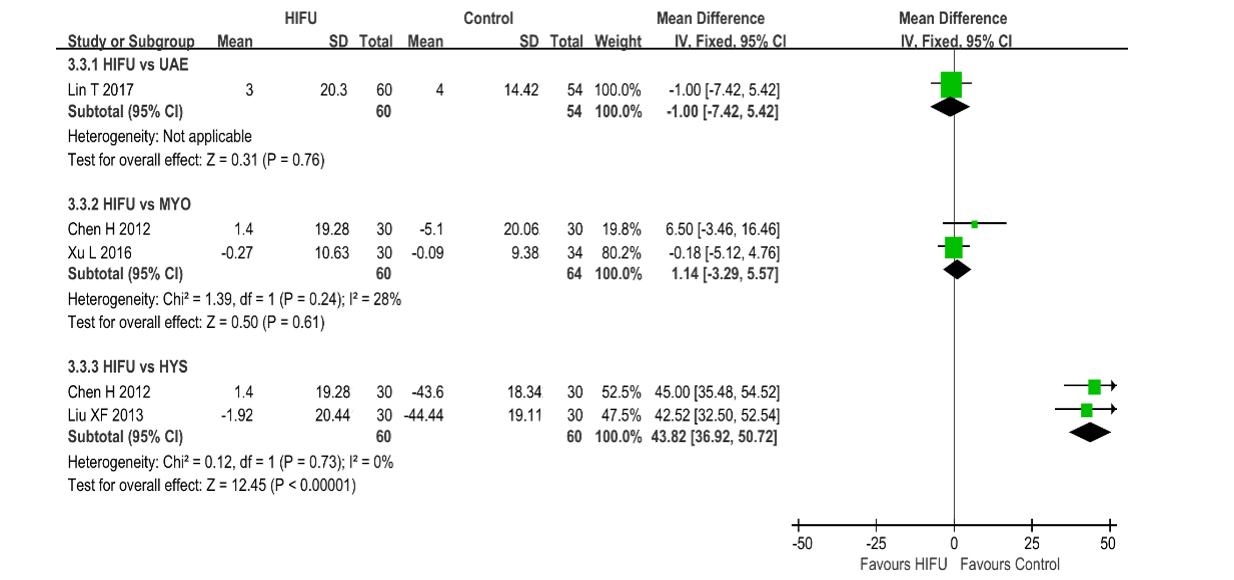

Supplement: Supplementary Figure 6 — Estradiol (E2) change of HIFU in comparison with other techniques. [file Image_6.TIF]

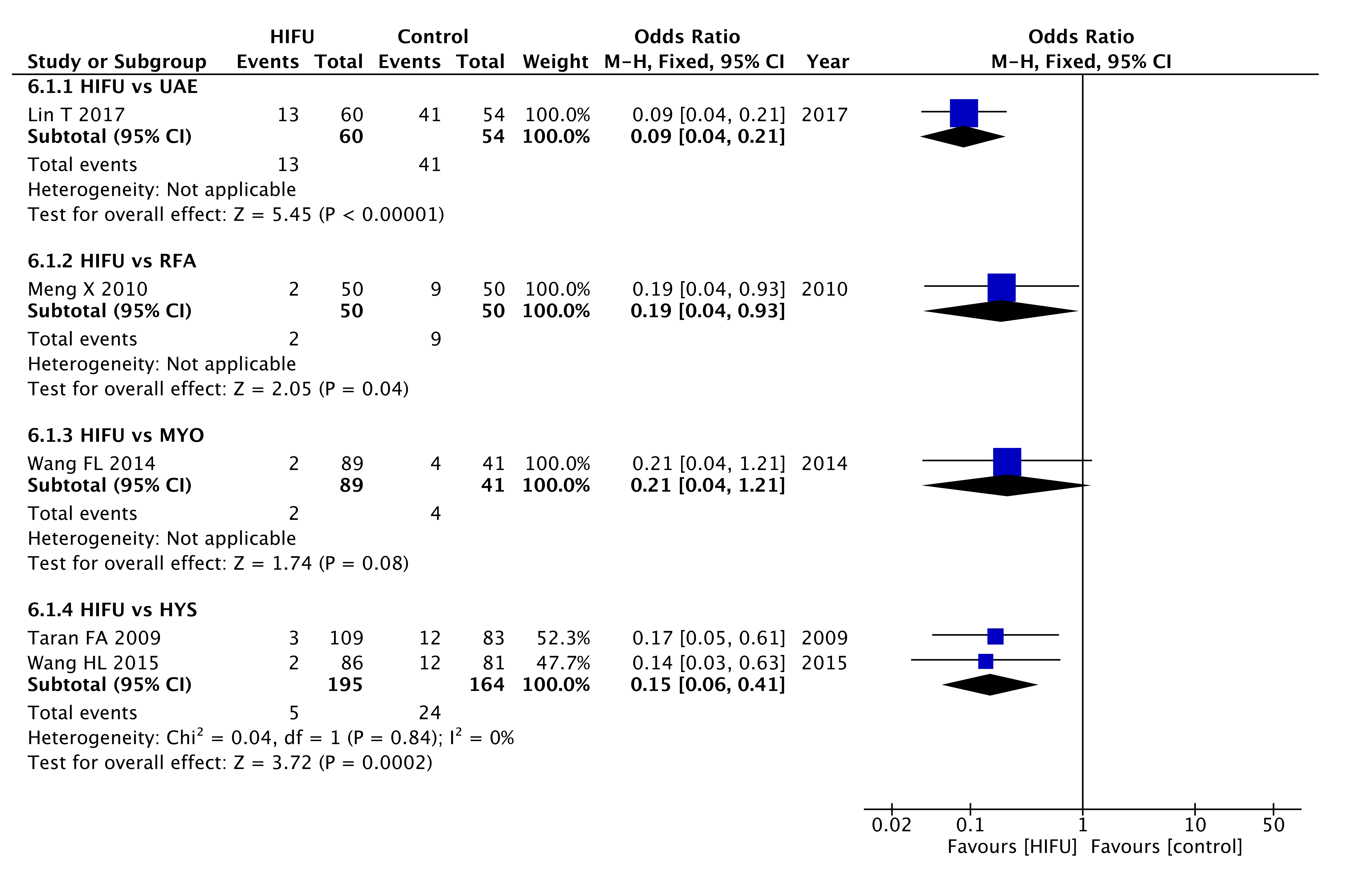

Supplement: Supplementary Figure 7 — Fever incidence of HIFU in comparison with other techniques: sensitivity analysis. [file Image_7.TIF]
