# Supplementary material for: Comparative Effectiveness and Safety of High-Intensity Focused Ultrasound for Uterine Fibroids: A Systematic Review and Meta-Analysis
Source: Front Oncol. 2021 Mar 9;11:600800. doi: 10.3389/fonc.2021.600800 (PMC7985460; doi:10.3389/fonc.2021.600800)
Supplement: Supplementary Table 1 — Risk of bias of included randomized controlled trials. [file Data_Sheet_1.docx]

**Table S1. Risk of bias of included randomized controlled trials**

| ID | Randomization | Allocation concealment | Blinding of outcome assessment | Incomplete outcome data | Selective reporting |
| --- | --- | --- | --- | --- | --- |
| Meng X 2010 | Unclear | Unclear | Low | Low | Low |
| Wang XY 2013 | Low | Unclear | Low | Low | Low |
| Li P 2018 | Low | Unclear | Low | Low | Low |
