# Supplementary material for: Comparative Effectiveness and Safety of High-Intensity Focused Ultrasound for Uterine Fibroids: A Systematic Review and Meta-Analysis
Source: Front Oncol. 2021 Mar 9;11:600800. doi: 10.3389/fonc.2021.600800 (PMC7985460; doi:10.3389/fonc.2021.600800)
Supplement: Supplementary Table 2 — Risk of bias of included non-randomized controlled trials. *For outcomes which were included in the systematic review. The items were scored as 0 (not reported), 1 (reported but inadequate), or 2 (reported and adequate). [file Data_Sheet_2.docx]

**Table S2. Risk of bias of included non-randomized controlled trials**

| ID | A clearly stated aim | Inclusion of consecutive patients | Prospective collection of data | Unbiased assessment of the outcomes* | Follow-up period appropriate to the outcomes* | Loss to follow up less than 5% | Prospective calculation of the study size | An adequate control group | Contemporary groups | Baseline equivalence of groups | Adequate statistical analyses | Total score |
| --- | --- | --- | --- | --- | --- | --- | --- | --- | --- | --- | --- | --- |
| Taran FA 2009 | 2 | 0 | 2 | 1 | 2 | 2 | 0 | 2 | 2 | 0 | 1 | 14 |
| Chen H 2012 | 2 | 0 | 2 | 1 | 1 | 2 | 0 | 2 | 2 | 2 | 2 | 16 |
| Liu XF 2013 | 2 | 0 | 2 | 1 | 2 | 2 | 0 | 2 | 2 | 2 | 2 | 17 |
| Wang FL 2014 | 2 | 0 | 2 | 0 | 2 | 1 | 0 | 2 | 2 | 1 | 1 | 13 |
| Wang HL 2015 | 2 | 0 | 2 | 0 | 2 | 2 | 0 | 2 | 2 | 2 | 2 | 16 |
| Xu L 2016 | 2 | 0 | 2 | 1 | 1 | 2 | 0 | 2 | 2 | 2 | 2 | 16 |
| Lin T 2017 | 2 | 0 | 2 | 1 | 2 | 2 | 0 | 2 | 2 | 2 | 1 | 16 |

*For outcomes which were included in the systematic review.

The items were scored as 0 (not reported), 1 (reported but inadequate) or 2 (reported and adequate)
