# Supplementary material for: Comparative Effectiveness and Safety of High-Intensity Focused Ultrasound for Uterine Fibroids: A Systematic Review and Meta-Analysis
Source: Front Oncol. 2021 Mar 9;11:600800. doi: 10.3389/fonc.2021.600800 (PMC7985460; doi:10.3389/fonc.2021.600800)
Supplement: Supplementary Table 3 — Risk of bias of included cohort studies. *For outcomes which were included in the systematic review. # Study controlled for: number of fibroid tumors/volume of all fibroid tissues or the maximal fibroid diameter, and age of patients. A study could be awarded a maximum of one star for each numbered item within the “Selection” and “Outcome” categories, and a maximum of two stars could be given for “Comparability.” [file Data_Sheet_3.docx]

**Table S3. Risk of bias of included cohort studies**

| ID | Selection | | | | Comparability of cohorts on the basis of the analysis^#^ | Outcome* | | | Number of stars |
| --- | --- | --- | --- | --- | --- | --- | --- | --- | --- |
|  | Representativeness of the exposed cohort | Selection of the non-exposed cohort | Ascertainment of exposure | Demonstration that outcome of interest was not present at start of study |  | Assessment of outcome | Was follow-up long enough for outcomes to occur | Adequacy of follow up of cohorts |  |
| Froeling V 2013 | ☆ | ☆ | ☆ | ☆ | - | ☆ | ☆ | - | 6 |
| Ikink ME 2014 | ☆ | ☆ | ☆ | ☆ | - | ☆ | ☆ | - | 6 |
| Barnard EP 2017 | ☆ | ☆ | ☆ | ☆ | ☆ | ☆ | - | - | 6 |
| Xiong XJ 2017 | ☆ | ☆ | ☆ | ☆ | ☆ | ☆ | ☆ | ☆ | 8 |
| Chen J 2018 | ☆ | ☆ | ☆ | ☆ | - | ☆ | ☆ | ☆ | 7 |
| Mohr-Sasson A 2018 | ☆ | ☆ | ☆ | ☆ | - | - | ☆ | - | 5 |
| Wu GP 2020 | ☆ | ☆ | ☆ | ☆ | ☆ | ☆ | ☆ | ☆ | 8 |
| Hu L 2020 | ☆ | ☆ | ☆ | ☆ | ☆ | ☆ | ☆ | ☆ | 8 |

*For outcomes which were included in the systematic review.

^#^ Study controlled for: number of fibroid tumors/volume of all fibroid tissue or the maximal fibroid diameter, and age of patients.

A study could be awarded a maximum of one star for each numbered item within the ‘Selection’ and ‘Outcome’ categories, and a maximum of two stars could be given for ‘Comparability’.
