# Supplementary material for: Comparative Effectiveness and Safety of High-Intensity Focused Ultrasound for Uterine Fibroids: A Systematic Review and Meta-Analysis
Source: Front Oncol. 2021 Mar 9;11:600800. doi: 10.3389/fonc.2021.600800 (PMC7985460; doi:10.3389/fonc.2021.600800)
Supplement: Supplementary Table 4 — GRADE evidence profile of the outcome of rate of re-intervention. 1. Cohort studies were assessed in accordance with the Newcastle-Ottawa Quality Assessment Scale (NOS), and the best quality of cohort studies should be those with nine stars. However, six stars were given to the three included studies. 2. The results from Barnard et al. (19), which only observed the short-term outcomes, did not find any statistical significant differences between HIFU and UAE. 3. Pooled odds ratio (OR) was 11.99 (95% confidence interval: 5.17–27.83, p < 0.001). 4. For Froeling et al. (13), volume of all fibroid tissues was significantly larger (p = 0.005) in the uterine artery embolization (UAE) group at baseline. However, in Ikink et al. (14), the maximum fibroid diameter and age at baseline were significantly higher (p < 0.005) in the MR-HIFU group. 5. Cohort studies were assessed in accordance with the NOS, and the best quality of cohort studies should be those with nine stars. Eight, seven, and five stars were given to the three included studies. 6. The results from Chen et al. (20) and Mohr-Sasson et al. (27) did not find any statistical significant differences between high-intensity focused ultrasound (HIFU) and myomectomy (MYO). 7. Pooled OR was 4.05 (95% CI 1.82–8.99, p < 0.001). 8. In Chen et al. (20), the mean age of the HIFU group was lower than that of the surgery group, and the uterine volume was smaller. In Mohr-Sasson et al. (27), women in the laparoscopic MYO group were younger (p < 0.001), and multiple uterine fibroid tumors were more common in the laparoscopic MYO group (p < 0.001). [file Data_Sheet_4.doc]

**Table S4. GRADE Evidence Profile of the outcome of Rate of re-intervention**

| **Quality assessment** | | | | | | | **No of patients** | | **Effect** | | **Quality** | **Importance** |
| --- | --- | --- | --- | --- | --- | --- | --- | --- | --- | --- | --- | --- |
|  |  |  |  |  |  |  |  |  |  |  |  |  |
| **No of studies** | **Design** | **Risk of bias** | **Inconsistency** | **Indirectness** | **Imprecision** | **Other considerations** | **Re-intervention** | **Control** | **Relative (95% CI)** | **Absolute** |  |  |
| **Re-intervention rate- HIFU vs UAE (follow-up 42 days to 5 years)** | | | | | | | | | | | | |
| 3 | observational studies | serious^1^ | serious^2^ | no serious indirectness | no serious imprecision | very strong association^3^ reduced effect for RR >> 1 or RR << 1^4^ | 44/130 (33.8%) | 8/149 (5.4%) | OR 11.99 (5.17 to 27.83) | 351 more per 1000 (from 173 more to 559 more) |  MODERATE | CRITICAL |
|  |  |  |  |  |  |  |  | 4.4% |  | 312 more per 1000 (from 148 more to 518 more) |  |  |
| **Re-intervention rate - HIFU vs MYO (follow-up 1 to 4 years)** | | | | | | | | | | | | |
| 3 | observational studies | serious^5^ | serious^6^ | no serious indirectness | no serious imprecision | strong association^7^ reduced effect for RR >> 1 or RR << 1^8^ | 34/1572 (2.2%) | 8/938 (0.9%) | OR 4.05 (1.82 to 8.99) | 25 more per 1000 (from 7 more to 63 more) |  LOW |  |
|  |  |  |  |  |  |  |  | 1% |  | 29 more per 1000 (from 8 more to 73 more) |  |  |

^1^ Cohort studies were assessed in accordance with Newcastle-Ottawa Quality Assessment Scale (NOS), and the best quality of cohort studies should be those with 9 stars. However, 6 stars were given to the three included studies.
^2^ The results from Bernard EP 2017, which only observed the short term outcomes, did not find any statistical significant differences between HIFU and UAE.
^3^ Pooled OR was 11.99 (95% CI 5.17 to 27.83, P<0.001).
^4^ For Froeling V 2013, volume of all fibroid tissue was significantly larger (P= 0.005) in the UAE group at baseline. However, for Ikink ME 2014, the maximum fibroid diameter and age at baseline was significantly higher (P<0.005) in the MR-HIFU group.
^5^ Cohort studies were assessed in accordance with Newcastle-Ottawa Quality Assessment Scale (NOS), and the best quality of cohort studies should be those with 9 stars. 8, 7, and 5 stars were given to the three included studies.
^6^ The results from Chen J 2018 and Mohr-Sasson A 2018 did not find any statistical significant differences between HIFU and MYO.
^7^ Pooled OR was 4.05 (95% CI 1.82 to 8.99, P<0.001).
^8^ For Chen J 2018, the mean age of the HIFU group was lower than that of the surgery group, and the uterine volume was smaller. For Mohr-Sasson A 2018, women in the laparoscopic myomectomy group were younger (P<0.001), and multiple uterine fibroid tumors were more common in the laparoscopic myomectomy group (P<0.001).
